# Draft Genome Sequences of *Neptuniibacter* sp. Strains LFT 1.8 and ATR 1.1

**DOI:** 10.1128/genomeA.01541-16

**Published:** 2017-02-02

**Authors:** A. L. Diéguez, J. L. Romalde

**Affiliations:** Departamento de Microbiología y Parasitología, CIBUS-Facultad de Biología, Universidade de Santiago de Compostela, Santiago de Compostela, Spain

## Abstract

We present the draft genomes of two strains previously identified as *Neptuniibacter* sp. LFT 1.8 (= CECT 8936 = DSM 100781) and ATR 1.1 (= CECT 8938 = DSM 100783) isolated from larvae of great scallops (*Pecten maximus*) and seawater, respectively. Both strains surely constitute two novel species in this genus, with putative applications for aromatic compound degradation.

## GENOME ANNOUNCEMENT

The genus *Neptuniibacter*, included in the family *Oceanospirillaceae*, was described by Arahal et al. (1) and is composed of only two species: *N. caesariensis* (type species of the genus) and *N. halophilus* (2), both isolated from marine environments. *Neptuniibacter* sp. CAR-SF has been studied because of its ability to degrade aromatic compounds such as carbazole (3) and *N. caesariensis* because of its release of sulfoacetate during the assimilation of taurine-nitrogen (4). The genome sequences of strains LFT 1.8 (= CECT 8936 = DSM 100781) and ATR 1.1 (= CECT 8938 = DSM 100783) were determined by Sistemas Genómicos (Valencia, Spain) using Illumina paired-end sequencing technology. The quality of reads was analyzed using Trimmomatic 0.32 (5). Genome assembly was performed using SPAdes 3.6.1 (6) and QUAST (7). The resulting genome for LFT 1.8 consists of 203 contigs (>200 bp) of 3,631,894 bp and had a G+C content of 45.7%. The *N*_50_ contig size is 285,345 bp, with the largest contig being 560,168 bp. For strain ATR 1.1, the genome has a size of 3,454,191 bp with 117 contigs (>200 bp) and a G+C content of 42.8%. The *N*_50_ contig size is 108,465 bp and the largest contig is 414,555 bp in length. Automatic gene annotation was carried out by the Rapid Annotations using Subsystems Technology (RAST) server (8), and tRNAs were identified by tRNAscan-SE v1.21 (9). The genome of LFT 1.8 contains 3,379 protein-encoding genes and 70 tRNAs, while ATR 1.1 contains 3,230 protein-encoding genes and 72 tRNAs.

The annotation of the genomes revealed the presence of the same 70 coding sequences related to resistance to antibiotics and toxic compounds in both strains, including resistance to vancomycin, fluoroquinolones, tetracycline, zinc, and arsenic or the presence of β-lactamase and mercuric reductase. In addition, the *Dmp* operon, which carries genes encoding enzymes for the degradation of phenol was detected. This cluster includes enzymes such as phenol hydroxylase (dmpK-P), catechol 2,3-dioxygenase, and 4-oxalocronate decarboxylase. Phenol is an important and toxic pollutant present in wastes of many industrial processes. To remove this compound, bacterial degradation is being investigated due to the considerable lower associated costs than physical-chemical methods.

There are six clustered regularly interspaced short palindromic repeat (CRISPR) arrays in the genome of strain LFT 1.8, including Cas1 and Cas3 helicase (*Yersinia* type) and Csy1-4 family proteins, but none in strain ATR 1.1.

The genome sequence of *Neptuniibacter* sp. LFT 1.8 and *Neptuniibacter* sp. ATR 1.1 and the curated annotation could contribute to a better understanding of their physiological and metabolic diversity and ecological functions. It could also open up new opportunities for the applications of these species to the degradation of aromatic compounds.

### Accession number(s).

These whole-genome shotgun projects have been deposited at DDBJ/ENA/GenBank under the accession numbers LUTR00000000 (*Neptuniibacter* sp. ATR 1.1) and LUTS00000000 (*Neptuniibacter* sp. LFT 1.8). The versions described in this paper are LUTR01000000 and LUTS01000000, respectively.
